# Drowning among older people: a neglected yet vital component of global drowning prevention

**DOI:** 10.1186/s40621-025-00651-4

**Published:** 2026-01-13

**Authors:** Ali Işın, Amy E. Peden

**Affiliations:** 1 Department of Coaching Education, Faculty of Sport Sciences, Akdeniz University, Antalya, 07058 Türkiye; 2 School of Population Health, Faculty of Medicine and Health, UNSW Sydney, Kensington, NSW 2052 Australia; 3 UNSW Beach Safety Research Group, UNSW Sydney, Kensington, NSW 2052 Australia

**Keywords:** Prevention, Aging population, Falls, Risk factors, Preventive interventions, Drowning

## Abstract

Globally in 2021, for the first time, the unintentional drowning fatality rate among people aged 70 years and older (8.15 per 100,000 people) surpassed the drowning rate of children under five years (7.66 per 100,000 people). While strong investment and advocacy in child drowning prevention have proven effective, we currently lack the research, consensus-based risk factors, and age-specific drowning prevention interventions for older people. Amid a globally aging population, we use this comment to highlight the need for increased evidence to reduce the persistent yet preventable fatal drowning rate for this age group.

Drowning is a preventable public health problem experienced in all regions of the world, although a disproportionate burden is recorded in low and middle-income countries. As a mechanism of injury, drowning may be either fatal or non-fatal in outcome [1] and can occur due to a variety of exposures including recreational interaction with water, the activities of daily life, in the act of migrating or due to occupational activities [2]. Legislative and community-based approaches can reduce drowning risk however, globally, implementation, enforcement and uptake vary dramatically [3, 4].

Globally, it is estimated that at least 3 million people have lost their lives to unintentional drowning over the past decade [4], though with the inclusion of drowning deaths due to water transport and flood-related drownings the true toll would be much higher. Of the 300,000 estimated unintentional drowning deaths worldwide in 2021, almost half (43%) were among children aged 14 years or younger [4].

Given this burden, the focus of the drowning prevention community has rightly been on the epidemiology, risk factors and strategies for the prevention of child drowning. Learning to swim is widely promoted as a drowning risk reduction strategy for school-aged children, while four-sided isolation fencing for swimming pools and formalised supervision via daycare, including the highly successful Anchal (creche) model in Bangladesh, have contributed to reductions in drowning fatalities among younger children [5, 6].

Although in many countries drowning rates remain highest among the paediatric population (i.e., children under five), globally there is a growing drowning burden among older people. According to the 2021 Global Burden of Disease (GBD) study, people aged 70 years and older were the age group with the highest rate of unintentional drowning (8.15 deaths per 100,000 people) in the world; surpassing children 0–4 years for the first time (7.66 per 100,000 people) (Fig. 1) [7]. Countries such as Japan, Thailand, Greece, and the Republic of Korea record extremely high drowning rates among people aged 70 years and over; in Japan’s case that rate is 3 times higher than the global average. Regionally, the Western Pacific, African, and South-East Asian regions are of particular concern [7]. As the world’s population ages and the number of older people increases [8], focused effort on a population that has been largely neglected in global drowning prevention is urgently required.

**Fig. 1:**
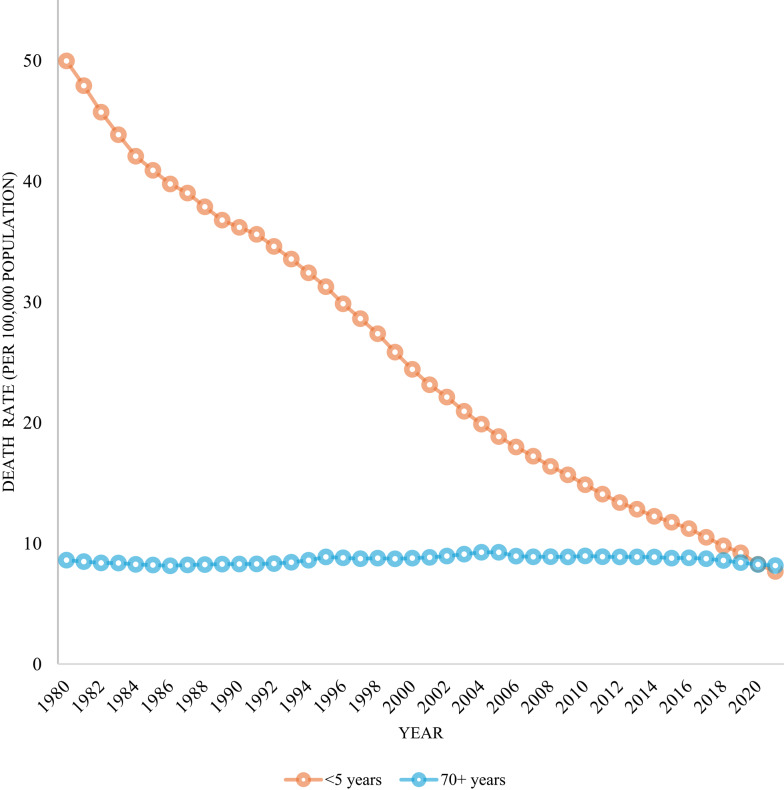
Global unintentional fatal drowning rates per 100,000 population for children 0–4 years and older adults 70 years and over, 1980 to 2021, Global Burden of Disease Study. *Source*: Redrawn using data from the Institute for Health Metrics and Evaluation (IHME). GBD Compare Data Visualization. Seattle, WA: IHME, University of Washington, 2024. Available from http://vizhub.healthdata.org/gbd-compare. (Accessed [11-09-2025])

Dedicated epidemiological studies of drowning risk among older people have historically been limited, resulting in a lack of consensus on how to define the older population among drowning prevention practitioners, the various stages of life within broad groupings of older age and the risk factors throughout this period [9]. It has been suggested that, declining physical ability, pre-existing co-morbidities (including dementia, sarcopenia, and cardiac conditions), polypharmacy, and alcohol consumption can contribute to drowning risk among older age groups [10]. Given the overlap with risk factors for falls [11], drowning prevention practitioners interested in reducing drowning risk among the older population may draw inspiration from the falls prevention effort such as learning from interventions targeting risk factors in home and community settings [12] and from collaboration within the fields of healthy aging and falls prevention [13].

Inspiration from other sectors will be necessary given the limited evidence on effective drowning prevention interventions for older people. Several of the World Health Organization’s (WHO) recommended strategies for preventing drowning address risk among the all-age population (e.g., build resilience and manage flood risks and other hazards, training bystanders in safe rescue and resuscitation and set and enforce safe boating regulations, including lifejacket wear) [14]. Due to historically high drowning risk among young children, many interventions focus on the pre-school and school age population, such as providing safe places away from water for pre-school children, installing barriers to control access to water and teaching school-aged children swimming and water safety skills. To date, what effectively reduces drowning risk among older populations remains unknown, with interventions predominantly having been proposed, rather than implemented or evaluated [9].

As a preventive health approach, physical activity is essential for healthy ageing, improving the performance of the activities of daily living, maintaining physical function and mobility, and reducing the risk of serious diseases and injuries, including drowning [15]. Aquatic activity has long been recommended for its low-impact nature but efficacious impact on cardiovascular, muscle, and bone health [16]. However, increased exposure to water increases the risk of drowning. Ceasing workforce participation can increase leisure time, affording more opportunity for aquatic activity. Older people should be encouraged to undertake pre-swim health checks. Such checks should provide counselling on the impact of pre-existing medical conditions and polypharmacy on drowning risk, as well as strategies to reduce that risk. Such strategies include the importance of swimming in supervised locations such as lifeguarded public pools or beaches. While such issues may be of relevance to reduce the risk of drowning for older people in high-income contexts, even less is known about drowning among older people in low and middle-income contexts. It is likely that a wide range of exposures precipitate drowning among the elderly in such contexts, including falls while walking near water, water transportation, occupational activities and being caught in floodwaters.

Drowning risk is not only associated with recreational activities in outdoor and natural bodies of water, but also in domestic settings, such as pools and bathtubs. Japan, as the country with the oldest population in the world, records the highest drowning rate in the world among its older adults [17], due in part to its in-home bathing culture. Strategies to reduce drowning risk while bathing include supervising older people with frailty during bathing, emptying the bathtub after use to prevent unintended entry and showering instead of bathing. Recognition of the intersection between drowning prevention and other sectors will be needed, such as proposing enhanced access to geriatric health services and nursing care facilities as a means of reducing bathtub drowning among the older population.

Drowning prevention and promoting safe aquatic activity intersects with many cross-cutting issues within the UN Decade of Healthy Ageing, such as disability, falls, physical activity, and rehabilitation [18]. Linked to physical health, aquatic activity also benefits mental health and water safety programs for older people can have the dual benefit of reducing drowning risk while combating social isolation and loneliness. Beyond health, the multisectoral nature of drowning and its prevention facilitates opportunities to embed the issue within broader agendas also addressing the wellbeing of older people [19] .

Since 2000, the global drowning death rate has fallen 38% [4]. As awareness of drowning and action towards preventing drowning grow, we cannot neglect the older subpopulation. As human lifespans extend, one in six people will be aged 60 years or older by 2030 [8]. It is of urgent importance that the issue of drowning among older people is fully considered by researchers, practitioners, policy makers and advocates to ensure we continue to maximise length and quality of life. Without timely and evidence-based action, underpinned by rigorous research to identify risk factors and determine effective prevention strategies, we risk a reversal of the positive progress made to date in reducing drowning.

## Data Availability

No datasets were generated during the current study. The dataset analysed (GBD study) is available online using the GBD compare tool.
